# Predicting amputation using machine learning: A systematic review

**DOI:** 10.1371/journal.pone.0293684

**Published:** 2023-11-07

**Authors:** Patrick Fangping Yao, Yi David Diao, Eric P. McMullen, Marlin Manka, Jessica Murphy, Celina Lin

**Affiliations:** 1 Michael G. DeGroote School of Medicine, McMaster University, Hamilton, ON, Canada; 2 Department of Computer Science, University of Western Ontario, London, ON, Canada; 3 Division of Physical Medicine and Rehabilitation, McMaster University, Hamilton, ON, Canada; 4 Division of Physical Medicine and Rehabilitation, Hamilton Health Sciences, Hamilton, ON, Canada; Air University, PAKISTAN

## Abstract

Amputation is an irreversible, last-line treatment indicated for a multitude of medical problems. Delaying amputation in favor of limb-sparing treatment may lead to increased risk of morbidity and mortality. This systematic review aims to synthesize the literature on how ML is being applied to predict amputation as an outcome. OVID Embase, OVID Medline, ACM Digital Library, Scopus, Web of Science, and IEEE Xplore were searched from inception to March 5, 2023. 1376 studies were screened; 15 articles were included. In the diabetic population, models ranged from sub-optimal to excellent performance (AUC: 0.6–0.94). In trauma patients, models had strong to excellent performance (AUC: 0.88–0.95). In patients who received amputation secondary to other etiologies (e.g.: burns and peripheral vascular disease), models had similar performance (AUC: 0.81–1.0). Many studies were found to have a high PROBAST risk of bias, most often due to small sample sizes. In conclusion, multiple machine learning models have been successfully developed that have the potential to be superior to traditional modeling techniques and prospective clinical judgment in predicting amputation. Further research is needed to overcome the limitations of current studies and to bring applicability to a clinical setting.

## Introduction

Amputation is an irreversible, last-line treatment indicated for several medical problems including trauma, peripheral vascular disease, diabetes, and cancer [1]. Delaying amputation in favor of limb-sparing treatment may lead to increased risk of morbidity and mortality [2]. On the other hand, due to the life-altering course of amputation, patients can experience a variety of complications, such as various psychological morbidities [3], phantom limb pain [4], and changes to patient self-esteem [3], following amputation [3, 5]. Patient quality of life is often severely decreased due to unique challenges related to mobility, social isolation, reduced energy, pain, sleep and emotional disturbance [6]. Given the substantial burden that can follow amputation, it is important for patients and providers to be aware of the likelihood of this outcome as early as possible to accept this inevitability, and to prevent undue morbidity and mortality through early amputation [7]. Determining the likelihood of amputation can help patients understand the importance of prophylactic changes that may help the patient avoid amputation.

Despite existing tools such as the Mangled Extremity Severity Score, accurately predicting amputation as an outcome is still a troublesome dilemma in many cases [8]. Correctly identifying the need for amputation throughout a patient’s disease course can improve outcomes, such as fewer postoperative complications (e.g.: decreased length of stay in hospital, fewer local ipsilateral limb complications while in hospital and fewer instances of unplanned revisions) [9]. Earlier identification of the need for amputation would also allow for a longer period of time to implement preoperative rehabilitation programs which could further improve postoperative outcomes [10]. There is also evidence to suggest that earlier identification can lead to a larger number of patients using prosthetics, and fewer ipsilateral leg complications that can worsen prosthetic use as well as worsen rehabilitation outcomes [11, 12]. Lastly, earlier prediction of amputation can aid multidisciplinary teams in providing emotional and psychological support well before the patient may receive surgery, thereby improving patient perception of the treatment decision [13]. Early prediction of amputation would ultimately allow patients to feel more involved with their decision-making process, which, in a systematic review, was found to lead to a better patient treatment experience [14].

Artificial intelligence (AI) is defined as a “machine-based system that can, for a given set of human-defined objectives, make predictions, recommendations, or decisions influencing real or virtual environments” [15]. These objectives are accomplished through having the AI “learn”, from datasets, the relationships that exists within the data. For instance, AI could review a dataset containing patient factors (e.g.: genetics, environment, patient vitals) and clinical outcomes, learn the relationships that exist, and use this information to predict future outcome in similar patients [16]. In a medical context, AI has been touted to be used in conjunction with electronic medical records (EMR) to help make medical predictions [17–19]. Machine learning (ML) is a subset of AI that uses prediction models and algorithms to analyze and draw inferences from patterns of data to learn or adapt. Machine learning is currently being used in a variety of ways ancillary to amputation, most of which have focused on patient outcomes after amputation [20–22]. There remains a gap in the literature about how ML has been applied to patient populations that may require amputation. This systematic review synthesized the literature to assess the status of ML with respect to prediction of amputation as an outcome.

## Methods

This systematic review was written in accordance with the PRISMA (Preferred Reporting Items for Systematic Reviews and Meta-Analyses) Checklist (S1 Checklist) and the R-AMSTAR (Revised Assessment of Multiple Systematic Reviews) guidelines for reporting systematic reviews. This study was registered in PROSPERO (registration number CRD42022375853).

### Eligibility criteria

#### Inclusion criteria

English peer-reviewed articles that developed multivariable models for predicting amputation in humans were included. No restriction on patient age was made.

#### Exclusion criteria

Publications were excluded if there was no mention of predicting amputation risk, or if AI was not a part of the methodology. Examples of this include AI models predicting only wound healing or outcomes rather than amputation. Abstracts from conferences were also excluded as they lacked depth and data to adequately contribute to the systematic review.

### Information sources

A systematic search of OVID Embase, OVID Medline, ACM Digital Library, Scopus, Web of Science, and IEEE Xplore from inception to November 12^th^, 2022, and re-updated on March 5^th^, 2023 with assistance from a medical librarian (S1 File). Studies resulting from this search were imported into Covidence, a systematic review software [23].

### Search strategy

A systematic review of the literature was completed using the subject heading “amputation” and the additional subject headings “machine learning”, "artificial intelligence”, “deep learning”. Numerous search terms were also used including “amputat*”, “AI”, “computer* assist* diagnos*”, “computer vision”, “neural network*”, “supervised learn*”, “unsupervised learn*”, “natural language process*”, “segmentat*”, and “reinforcement learn*” (S1 File). The search terms “predict*” and “risk” were not included to broaden the search. The references of included articles were checked manually for citation chaining. All literature (interventional, observation, and otherwise) were eligible for inclusion during the initial screening. The literature from the search was screened based on their title and abstract. Duplicates were removed, and those that met the inclusion criteria progressed to the full-text screening stage for more in-depth screening.

Two reviewers (P.Y., Y.D.) completed the title and abstract review screening for eligible studies independently and in duplicate. A full-text review was subsequently conducted. Data was extracted independently and duplicate and discrepancies at each stage were resolved through review with a third author (E.M.). Risk of bias of each study was assessed using the PROBAST Risk of Bias for Predictive Models assessment tool and given either low, high, or unclear designations as outlined [24]. The authors considered Newcastle (in PROSPERO protocol), however, PROBAST was ultimately favored given the superior applicability of assessing risk of bias in machine learning models.

### Data charting and result reporting

Data was extracted from the 15 included articles into a data extraction table created a priori. The following pre-selected variables were extracted from each included article: author(s), year of publication, country of dataset origin, study design, level of evidence, primary aim(s), secondary aim(s), ML model(s) used, derivation/validation test used, reference test used, comparison to reference, secondary reference test used (if applicable), comparison to the secondary reference test, clinical applicability of the ML model, dataset, study inclusion criteria, study exclusion criteria, underlying pathology, anatomical part being studied, number of patients in dataset, the number of cases in data set, sex (Female, Male [%]), age (range), how the model was trained, features in the model, predictors of amputation, performance metric used, study limitations, conclusion(s), any notes made by the authors of this systematic review, and any conflicts of interest.

## Results

**Fig 1:**
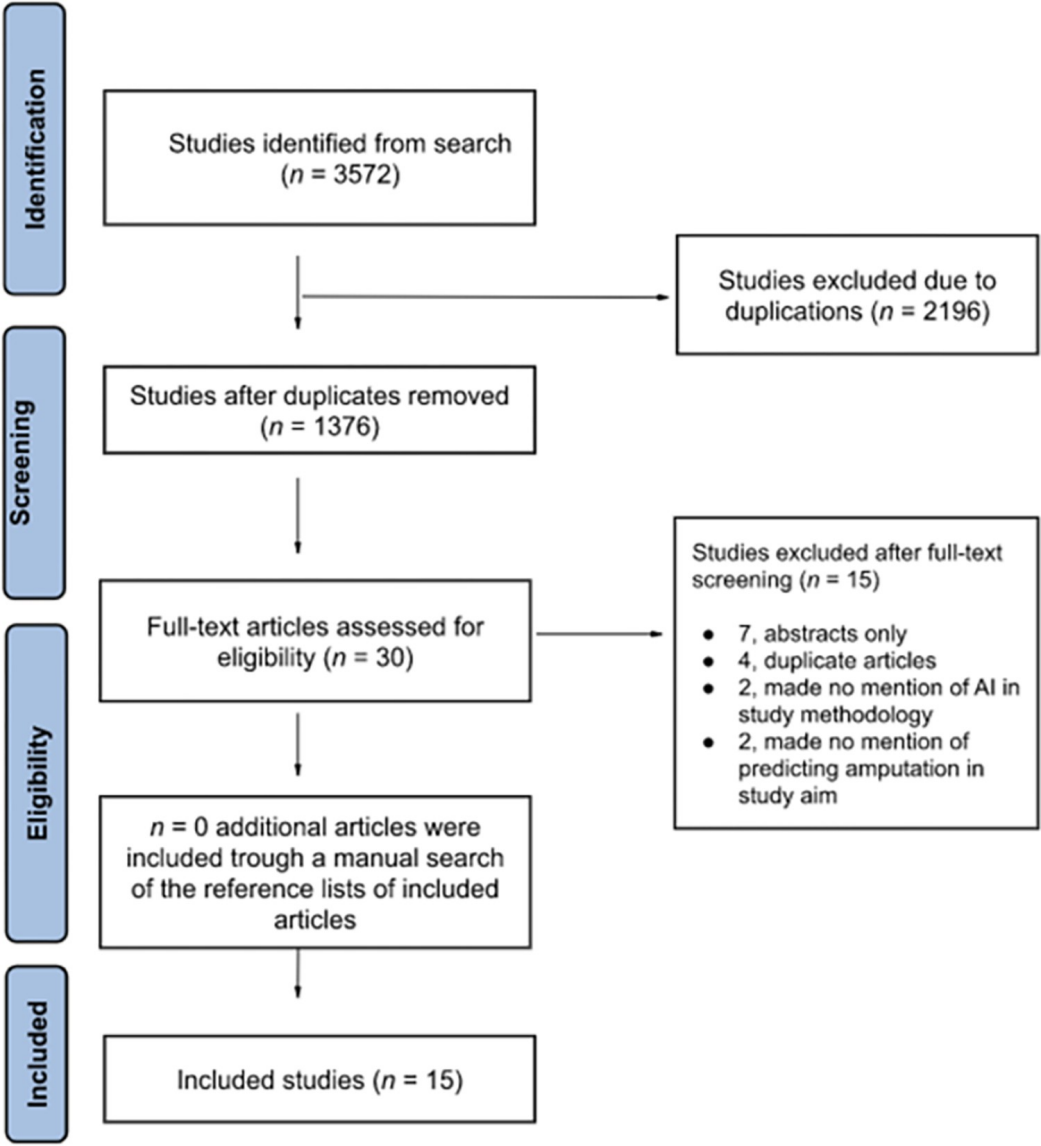
Flow diagram of literature screening using the Preferred Reporting Items for Systematic Reviews and Meta-Analyses (PRISMA) guidelines [42].

The search yielded 3572 articles; after duplicates were removed, 1376 articles remained and underwent title and abstract screening. Thirty articles moved through to full-text review, with 15 of these meeting the criteria for inclusion in this systematic review (Fig 1). The included studies developed and validated ML models from a total of 2,261,790 patients. Extensive heterogeneity between the studies across study objectives, ML models, data set features, varying subgroup analyses, and performance metrics of included studies precluded a meta-analysis of such findings. The performance metric in the majority of included articles [25–36] was the area under the curve of the receiver operator characteristic (AUC) which is standard for the evaluation of application of ML in medical contexts [37, 38]. However, three studies used other performance metrics, including F-score (Fβ) [31], out of bag error rate [39], or only accuracy [40]. For simplicity of reporting, the included studies were categorized by amputation etiology. Most studies reported on patients who received amputation due to Diabetes [25–29, 35, 36, 39–41], followed by Trauma [30–32], and “Other” [33, 34]. All included studies were derivation studies that included a form of validation. 12 of the included studies performed only internal validation, while the remaining three [33–35] included external validation as well.

### Diabetes

Table 1 shows those studies that applied ML-based prediction models for patients with diabetes. The variables that were found to be important features in models varied. However, some of these variables, including increased age, Wagner scores, C-reactive protein and history of amputation among others, appeared in multiple models [26–29, 36, 39–41]. The models within these studies ranged in performance from sub-optimal to excellent [AUC: 0.6–0.94]. Random Forest models [25, 28, 29, 35, 36], Gradient Boosted [27, 28], and Logistic Regression [25, 29, 35, 39] were used in multiple studies as the modeling technique. Only two of the studies had comparison reference tests to a non-ML prediction model [27, 40]. In those studies, the ML-based prediction model had better performance than the non-ML-based prediction model. Two of the studies [29, 36] produced online tools aimed at helping clinicians stratify the risk of amputation based on their modelling. Only five of the included studies [27, 28, 32, 33, 35] could be classified as low risk of bias according to the PROBAST Risk of Bias assessment tool. Within these studies, a history of amputation, age, and diabetic complications such as peripheral vascular disease or kidney complications were features that appeared useful in more than one of the models for prediction of amputation. One study [27] was rated as having an unclear risk of outcome due to the use of basket error rate as the sole performance metric as well as having unclear methodology in derivation and validation of their model.

**Table 1:** Summary of models predicting amputation in research studying diabetes.

Author, year, country	Underlying Pathology	ML model(s) studied	Variables identified by ML model	Performance metric(s) of best performing model	PROBAST Risk of Bias
Austin et al., 2022, USA [39]	Diabetes with Peripheral artery disease	LR	Foot Exam, HbA1C, Vascular Image, Urban, Comorbidity Count, Medicare-Medicaid Dual Eligible, White, Female, Age	Out-Of-Bag Error Rate—31%	Unclear
Du et al., 2022, China [25]	Diabetic Foot Ulcers	LR SVM RF GBDT ANN XGBoost	2019 data: white blood cells, blood potassium, and prehospital delay, 2020 data: prehospital delay, ischemia, and serum albumin	**XGBoost**: AUC—0.86, Accuracy—0.8, Sensitivity—0.67, Specificity—0.86, PPV—0.67, NPV—0.86	High
Kasbekar et al., 2017, India [40]	Diabetic Foot	C5.0 (Simple and Boosted Ensemble)	**Simple:** Doppler status of flow in the affected limb, Wagner DFU staging **Boosted Ensemble:** Days admitted, Ulcer grade: 1–5, Normal Foot X-ray normal doppler, monophasic doppler, biphasic doppler, triphasic doppler, HbA1c, creatinine, duration of symptoms, presence of other comorbidities, age, prothrombin time, BSL on admission, diabetes, albumin, negative culture report, hemoglobin, bilirubin	**Boosted Ensemble**: Accuracy—0.96, Kappa—0.92	High
Lin et al., 2020, Not Reported [26]	Diabetic Foot	BPNN	Platelet, hemoglobin, apolipoprotein A1, total white blood cell count, alkaline phosphatase, and severe ulcer conditions, glycated hemoglobin, low-density lipoprotein cholesterol, high-density lipoprotein cholesterol, dorsal artery pulsation, and hypersensitive C-reactive protein	**BPNN**: AUC—0.924, Sensitivity—1, Specificity—0.8182	High
Ravaut et al., 2021, Canada [27]	Diabetes	GBDT	Age, History of Amputation, History of adverse outcomes from diabetes complications, Area-level ethnic concentration quintile, landing date for immigrants, diagnostic radiology billing in the last two years (spread), history of emergency department visits, average time between consecutive dispensed prescriptions in the last two years	Average test AUC—0.6894 (0.689–0.692)	Low
Schäfer et al., 2021, Denmark [35]	Diabetes	LR RF classifiers	Diabetes and cardiovascular, peripheral artery disease, neuropathy, and chronic renal complications	AUC is presented in ROC curve format without specific values. LR showed better performance in the ROC curves for prediction of amputation within 2 years, 3 years and 5 years.	Low
Stefanopoulos et al. 2022, USA [36]	Diabetic Foot Ulcers	LASSO Regression RF	Gangrene, Septic Shock, Peripheral vascular disease, weight loss, septicemia, systematic infection, Anemia, Age>40, Bactermia, elective procedure	**10 Variable LASSO**: AUC—0.84, Performance—0.778, Specificity—0.79, Sensitivity—0.767	Low
Xie et al., 2022, China [41]	Diabetic Foot Ulcers	LGBM	Higher Wagner score, WIfI score or gangrene	Weighted-average AUC—0.90, Sensitivity—0.871, Specificity—0.744, NPV—0.797 and PPV—0.863	High
Wang et al. 2022, China [29]	Texas Grade 3 Diabetic Foot Ulcers	Decision Tree SVM XGBoost, LR RF	Random blood glucose, years with diabetes, cardiovascular disease, peripheral arterial disease, smoking history, albumin, serum creatinine, C-reactive protein, and DFU history	**XGBoost**: AUC—0.881, Accuracy—0.814, Precision—0.846, Recall—0.767, F1 Score—0.805	High
Yang et al., 2021, USA [28]	Diabetes and taking canagliflozin	LASSO GBM Elastic net RF	**LASSO**: History of LEA, Use of Loop Diuretics, Use of Sulfonylureas. Female, Use of thiazolidinediones, depression, anemia, race (Black), hyperlipidemia, Use of DPP4 inhibitors, prostatic hyperplasia, use of insulin, age (Years), hypertension, acquired hyperthyroidism, rheumatoid arthritis/osteoarthritis **GBM**: age, diabetes duration, prostatic hyperplasia, female sex, history of LEA, asthma, rheumatoid arthritis osteoarthritis, use of sulfonylureas, and use of loop diuretics	**LASSO**: AUC—0.81 (95% CI: 0.76–0.86), Specificity—0.5830, PPV—0.0131, NPV—0.9977	Low

LR: Logistic regression; SVM: Support vector machine; RF: Random forest; GBDT: Gradient boosting decision tree; ANN: Artificial neural network; XGBoost: Extreme gradient boosting algorithm; AUC: Area under the receiver operator curve; PPV: Positive predictive value; NPV: Negative predictive value; DFU: Diabetic foot ulceration; BPNN: Back propagation neural network; LR: Logistic regression; LASSO: Least absolute shrinkage and selection operator-type regularized regression; LGBM: Light gradient boosted machine WIfI: Wound, Ischemia, and foot Infection classification system; LEA: Lower extremity amputation

### Trauma

Table 2 shows the studies that used a ML-based prediction model for patients who had suffered physical trauma. All the studies for this population showed strong to excellent performance (AUC: 0.88–0.95). Each of these studies used different base ML learning models. Two studies [31, 32] looked at lower extremity injury with concurrent vascular injury and shared the same predictor variable of arterial injury. Bevevino et al. [30] compared their model to a non ML-based model, with theirs resulting in better performance. Perkins et al.’s [32] model was rated “Unclear” in the applicability section of the PROBAST score as they tested for the chance of revascularization and limb viability and did not directly test for amputation as an outcome. In addition, the population in both the derivation and validation of their model was 100% military personnel, therefore, their model may not be generalizable to other populations [32]. Perkins et al. [32] compared their results with those determined with the Mangled Extremity Severity Score (MESS), a clinical decision-making tool created in 1990 and validated in 2001 [2, 43]. Perkins et al. [32] demonstrated that their Supervised Bayesian Network model showed better performance in predicting the revascularization of limbs.

**Table 2:** Summary of models predicting amputation in research studying trauma.

Author, year, country	Underlying Pathology	ML model(s) studied	Variables identified by ML model	Performance metric(s) of best performing model	PROBAST Risk of Bias
Bevevino et al., 2014, USA [30]	Open calcaneus fractures	ANN	American Society of Anesthesiologist grade, plantar sensation, fracture treatment before arrival, Gustilo-Anderson fracture type, Sanders fracture classification, vascular injury, male sex, dismounted blast mechanism	AUC—0.8 (95% CI: 0.77, 0.82), Sensitivity 0.64, Specificity– 0.91, PPV– 0.86, NPV– 0.74	High
Bolourani et al., 2021, USA [31]	Traumatic lower extremity injury with concomitant vascular injury	RF XGBoost Supervised LR classifier-based model	APRDRG mortality risk, APRDRG severity of Illness, Length of stay Thrombocytopenia, Leukocytosis, Above knee arterial injury, Open fracture, External fixation, Timing of lower extremity bypass during initial admission, Timing of lower extremity bypass on readmission	**LogisticReg**—AUC—0.88, AUCPR—0.71, Accuracy—0.88, Sensitivity—0.47, Specificity—0.98, G mean—0.71, Fβ Score—0.48	High
Perkins et al., 2020, USA + United Kingdom [32]	Lower-extremity arterial injury	Supervised Bayesian Network	Mechanism of injury, Arterial injury (anatomical level, multiple level, number of tibial arteries injured), Associated injury (soft tissue injury, fracture), complications (shock, duration of ischemia, compartment syndrome), method of arterial repair	Internal Val: AUC—0.95 (95% CI: 0.93–0.98), Sensitivity—0.946, Specificity– 0.85, Diagnostic Odds ratio– 0.985 External Val: AUC 0.97 (95% CI: 0.95–1)	High

ANN: Artificial neural network; AUC: Area under the receiver operator curve; PPV: Positive predictive value; NPV: Negative predictive value; RF: Random forest; XGBoost: Extreme gradient boosting algorithm; LR: Logistic regression; APRDRG: All Patients Refined Diagnosis Related Groups; Val: Validation

### Other

Table 3 shows the studies that used ML-based prediction models for all other pathologies. Two studies [33, 34] were included, Cox et al. was classified as low PROBAST risk of bias. Models from both studies [33, 34] used the random forest ML model, and they both had a strong performance (AUC:0.81–1.0). Martinez-Jimenez et al. [34] demonstrated the applicability of their ML model in a cohort of 22 prospective burn patients, correctly identifying all patients that would later go on to require amputation by the surgeon’s independent decision. Uniquely, this study was the only one of the 15 included studies that analyzed imaging, using thermograms to assess and delineate the patients [35].

**Table 3:** Summary of findings from models studying other etiologies.

Author, year, country	Underlying Pathology	ML model(s) studied	Variables identified by ML model	Performance metric(s) of best performing model	PROBAST Risk of Bias
Cox et al., 2022, USA [33]	Peripheral arterial disease	RF	elective surgery designation, claudication, open wound or wound infection, white blood cell count, serum albumin, INR, major reintervention of a treated arterial segment, systemic sepsis, renal failure, and hematocrit.	Internal Val: AUC—0.81 (95% CI: 0.78, 0.85), Accuracy—0.74, Sensitivity—0.74, Specificity—0.70 External Val: AUC– 0.81 (95% CI: 0.76–0.86)	Low
Martínez-Jiménez et al., 2018. Mexico [34]	Burns	Recursive partitioning RF and unsupervised k-means clustering	ΔT >5.0˚C	Internal Val: AUC—1.0, Sensitivity—1, Specificity—1, PPV—1, NPV 1 External Val: weighted kappa—0.901	High

RF: Random forest; AUC: Area under the receiver operator curve; ΔT: Temperature difference; PPV: Positive predictive value; NPV: Negative predictive value; Val: Validation

## Discussion

Amputation is a life-altering, but often necessary procedure resulting from consequences associated with conditions such as diabetes or limb trauma. Proper early identification of the need to amputate can help mitigate negative outcomes associated with amputation [2], and provide patients with the appropriate time to prepare for the potential physical and emotional or psychological complications that can follow the intervention [2, 3, 5, 6]. Earlier work has used AI and ML to make medical predictions, including predicting outcomes following amputation. Researchers have also been attempting to create ML models to predict factors associated with the outcome of amputation. It is difficult to understand the potential use of ML in predicting amputation as an outcome, as there has been no published review of these studies until now. This systematic review aimed to synthesize the available literature using ML to predict amputation. Results demonstrated the potential for ML to predict amputation as an outcome across multiple different target populations. Most of the studies in this review were able to produce predictive models with good performance, with some demonstrating improved sensitivity and specificity compared to non-ML prediction models or clinical decision-making tools. In addition, Martínez-Jiménez et al. showed comparability to clinical decision-making in a prospective setting, a requirement for the future implementation of ML in medicine [34]. Collectively, the results of this review showcase the viability of ML modeling in creating predictions for amputation. These models could be used to accurately forecast the clinical course of a patient and inform clinicians on personalized treatment plans including interventional or prophylactic changes.

Despite the promising nature of the results, there are several limitations that should be considered. The first of these arises from the review process itself. Only full-text, peer-reviewed articles published in English were included in this study. This likely resulted in an overrepresentation of research from primarily English-speaking countries. Furthermore, there are limitations to the studies themselves. The results demonstrate heterogeneity between the features that were important to predict amputation. This could be due to the variance in the data fields between the datasets, the discrepancies between each modeling technique, the intrinsic reliability of the models themselves, or any combination of these factors. Many of the datasets that were used for derivation were pre-existing databases, therefore restricting the variables that could be collected and analyzed between models. In addition, many of the studies discussed the database fields’ restrictions, arguing that the granularity within variables such as surgery outcomes and the severity of disease or injuries can be limiting in many of the datasets [31, 33, 35]. The inconsistency between database variable recording can therefore alter the impact these features could have between models or if they were to feature in a model at all. Taken together, these variations limit the confidence that can be placed in any trends or correlations that may be observed in important features across models and studies. Furthermore, despite the independent models demonstrating positive numbers, one cannot synthesize a summative conclusion from their amalgamation. The non-uniformity in outcomes such as the window of consideration for the outcomes limits the ability to compare [30, 31]. In addition, the applicability to the study population was variable across studies, with some studies deriving their models from a cohort sharing a specific trait that would limit generalizability to the other patient populations [28, 32, 33], and others limited by having no external validation [27, 28, 32, 34]. Lastly, a large number of the studies had a high risk of bias owing to small sample sizes [25, 26, 30, 31, 40, 41], therefore resulting in the need for further validation both internally and externally.

Given the current work done with ML and amputations, the results show the potential for ML to be clinically impactful. Although some authors provided online tools produced from their models [30, 37], the overall reliable application of the current models studied is limited. Increasing the breadth of data collected and standardizing the outcome measures would help to mitigate the heterogeneity seen across variables considered between models. Ultimately, despite the evidence that these models can be developed to accurately predict outcomes, for these models to build credibility, more studies that have a low risk of bias must be produced. These then need to be taken into clinical settings to study the validity and utility of these models or tools in each cohort. Lastly, future research that investigates the outcomes of change in management in cohorts applying ML based risk stratification should be pursued. The results of interventions such as increased surveillance and education in patients who are classified as higher risk for amputation should be clarified to understand the true extent of the impact that predicting amputation early will have.

In conclusion, this systematic review shows that multiple ML models with various target populations have been successfully derived that have the potential to be superior to traditional modeling techniques and comparable to prospective clinical judgment. Despite existing clinical decision-making tools, being able to accurately predict amputation as an outcome is a clinical question that has yet to be conclusively answered. There is notable interest in the applications of AI in this area, a body of research growing particularly in the last decade. Despite the promise, there are several limitations stalling the growth of these modeling technologies in a clinical context including heterogeneity between database variables and therefore model features, and bias or lack of applicability in the derivation and validation of the models themselves. Although clinical decision making tools based on these models are starting to be created, future research is needed that includes more robust databases designed to validate ML models against external cohorts in order to confidently apply this technology in clinical settings.

## Supporting information

S1 Checklist Prisma checklist. (DOCX)

S1 File Search strategy. (DOCX)

S1 Dataset Extractions. (XLSX)
