# Supplementary material for: Predicting amputation using machine learning: A systematic review
Source: PLoS One. 2023 Nov 7;18(11):e0293684. doi: 10.1371/journal.pone.0293684 (PMC10629636; doi:10.1371/journal.pone.0293684)
Supplement: S1 File — (DOCX) [file pone.0293684.s002.docx]

S2. Search Strategy

**Database(s):**
Embase <1974 to 2023 March 03>

| **#** | **Query** | **Results from 5 Mar 2023** |
| --- | --- | --- |
| 1 | exp artificial intelligence/ | 77,081 |
| 2 | exp machine learning/ | 385,194 |
| 3 | deep learning/ | 38,937 |
| 4 | ((artificial* or machine* or deep*) adj3 (intelligence or learning)).tw,kw. | 153,675 |
| 5 | AI.ti,ab. | 56,955 |
| 6 | computer* assist* [diagnosis.tw](http://diagnosis.tw/),kw. | 1,142 |
| 7 | computer [vision.mp](http://vision.mp/). | 8,621 |
| 8 | supervised learn*.mp. | 5,982 |
| 9 | neural network*.mp. | 130,272 |
| 10 | unsupervised learn*.mp. | 2,893 |
| 11 | natural language process*.mp. | 11,773 |
| 12 | segmentat*.mp. | 80,075 |
| 13 | reinforcement learn*.mp. | 6,734 |
| 14 | amputat*.mp. | 80,058 |
| 15 | exp amputation/ | 55,759 |
| 16 | 1 or 2 or 3 or 4 or 5 or 6 or 7 or 8 or 9 or 10 or 11 or 12 or 13 | 573,129 |
| 17 | 14 or 15 | 80,292 |
| 18 | 16 and 17 | 580 |

Ovid MEDLINE(R) and Epub Ahead of Print, In-Process, In-Data-Review & Other Non-Indexed Citations, Daily and Versions <1946 to March 03, 2023>

| **#** | **Query** | **Results from 5 Mar 2023** |
| --- | --- | --- |
| 1 | exp artificial intelligence/ | 167,332 |
| 2 | exp machine learning/ | 54,735 |
| 3 | deep learning/ | 14,637 |
| 4 | ((artificial* or machine* or deep*) adj3 (intelligence or learning)).tw,kw. | 123,571 |
| 5 | AI.ti,ab. | 41,157 |
| 6 | exp computer assisted diagnosis/ | 86,544 |
| 7 | computer* assist* [diagnosis.tw](http://diagnosis.tw/),kw. | 782 |
| 8 | computer [vision.mp](http://vision.mp/). | 7,396 |
| 9 | supervised learn*.mp. | 4,953 |
| 10 | neural network*.mp. | 101,426 |
| 11 | unsupervised learn*.mp. | 2,429 |
| 12 | natural language process*.mp. | 10,023 |
| 13 | segmentat*.mp. | 52,156 |
| 14 | reinforcement learn*.mp. | 5,700 |
| 15 | exp Amputation, Surgical/ | 24,164 |
| 16 | amputat*.mp. | 58,939 |
| 17 | (1 or 2 or 3 or 4 or 5 or 6 or 7 or 8 or 9 or 10 or 11 or 12 or 13 or 14) and (15 or 16) | 377 |

**ACM Digital Library:** 993 results
amputat* AND (artificial intelligence OR machine learning OR deep learning OR AI OR computer* assist* diagnos* OR computer vision OR supervised learn* OR neural network unsupervised learn* OR natural language process* OR segmentat* OR reinforcement learn*)

**Scopus:** 607 results
“amputation” AND (“artificial intelligence” OR “machine learning” OR “deep learning” OR “AI“ OR “computer assisted diagnosis” OR “computer vision” OR “supervised learning” OR “neural network” OR “unsupervised learning” OR “natural language processing” OR “segmentation” OR “reinforcement learning”)

**IEEE Xplore:** 190 results
amputat* AND (artificial intelligence OR machine learn* OR deep learn* OR AI OR computer* assist* diagnos* OR computer vision OR supervised learn* OR neural network* OR unsupervised learn* OR natural language process* OR segmentat* OR reinforcement learn*)

**Web of Science:** 825 results
amputat* AND (artificial intelligence OR machine learn* OR deep learn* OR AI OR computer* assist* diagnos* OR computer vision OR supervised learn* OR neural network* OR unsupervised learn* OR natural language process* OR segmentat* OR reinforcement learn*)

The following amendments to information provided in the PROSPERO protocol were made: PROBAST was used as opposed to GRADE for assessing Risk of Bias due to PROBAST’s applicability for predictive models. The search was initially completed November 11^th^, 2022, however, a search was re-done on March 5^th^, 2023 with the guidance of a medical librarian.

The authors of this systemic review will do their best in ensuring all data relevant to this systemic review is publicly available with no restrictions.
